# Body mass index is associated with clinical outcomes in idiopathic pulmonary fibrosis

**DOI:** 10.1038/s41598-024-62572-4

**Published:** 2024-05-24

**Authors:** Hee-Young Yoon, Hoseob Kim, Yoonjong Bae, Jin Woo Song

**Affiliations:** 1 Division of Allergy and Respiratory Diseases, Soonchunhyang University Seoul Hospital, Seoul, Republic of Korea; 2 Department of Data Science, Hanmi Pharm. Co., Ltd, Seoul, Republic of Korea; 3 Department of Pulmonary and Critical Care Medicine, Asan Medical Center, University of Ulsan College of Medicine, 88, Olympic-Ro 43-Gil, Songpa-Gu, Seoul, 05505 Republic of Korea

**Keywords:** Lung, Body weight, Obesity, Mortality, Hospitalization, Respiratory tract diseases, Epidemiology

## Abstract

Association between body mass index (BMI) and prognosis in patients with idiopathic pulmonary fibrosis (IPF) remains uncertain. We investigated the association between BMI and clinical outcomes in patients with IPF using national health claims data. The study included 11,826 patients with IPF and rare incurable disease exemption codes (mean age: 68.9 years, male: 73.8%) and available BMI data who visited medical institutions between January 2002 and December 2018. Multivariable Cox proportional hazard models were used to evaluate the association of BMI with all-cause mortality and hospitalization. Based on BMI, 3.1%, 32.8%, 27.8%, and 36.4% were classified as underweight, normal, overweight, and obese, respectively. Multivariable analysis showed independent associations of overweight (hazard ratio [HR] 0.856, 95% confidence interval [CI] 0.801–0.916) and underweight (HR 1.538, 95% CI 1.347–1.757) with mortality in patients with IPF. Similarly, overweight (HR 0.887, 95% CI 0.834–0.943) and underweight (HR 1.265, 95% CI 1.104–1.449) were also associated with hospitalization in patients with IPF in the multivariable analysis. Spline HR curve analysis adjusted for all covariates revealed a non-linear relationship between BMI and mortality in patients with IPF. Our data suggest that BMI is associated with clinical outcomes in patients with IPF.

## Introduction

Idiopathic pulmonary fibrosis (IPF) is a chronic progressive fibrosing interstitial pneumonia with unknown etiology and is characterized by progressive dyspnea and declining lung function^1^. Patients with IPF have poor prognosis and without treatment, a median survival of 3–5 years after diagnosis^2–4^. Several prognostic factors in IPF have been proposed, including older age, male sex, lower body mass index (BMI), impaired lung function, decreased exercise capacity, greater extent of fibrosis on chest computed tomography, and the presence of comorbidities such as emphysema and pulmonary hypertension^2,4–8^. BMI, an anthropometric index of height and weight, serves as a surrogate marker for body fat mass. In the general population, there is a J-shaped association between BMI and overall mortality^9^. BMI < 25.0 kg/m^2^ is inversely associated with mortality. Conversely, BMI > 25.0 kg/m^2^ is positively associated with mortality^9,10^.

However, previous studies have demonstrated a positive correlation between higher BMI and improved prognosis in patients with IPF^6–8,11–17^. During a median follow-up period of 20 months, in a study involving 138 patients with IPF, a low BMI (< 30.0 kg/m^2^) was associated with a higher overall mortality (35% vs. 20%, p = 0.017) than a high BMI (≥ 30.0 kg/m^2^)^7^. In the Japanese IPF cohort (n = 14,783), in-hospital mortality risk was highest in the underweight group (< 18.5 kg/m^2^) and lowest in the obese group (≥ 30.0 kg/m^2^)^8^. A study from the United States that included 197 patients with IPF showed, in a multivariable analysis adjusted for clinical variables, that a lower BMI was associated with a higher mortality risk (hazard ratio [HR] 0.86; 95% confidence interval [CI] 0.79–0.94)^6^. However, most of these studies have a relatively small sample size (n = 46–1061) from a single center and have focused solely on the simple linear relationship between BMI and prognosis^6,13,14^. Therefore, our study aimed to investigate the association between BMI and prognosis in a large nationwide cohort of patients with IPF.

## Materials and methods

### Data sources

Data for this study were collected from the National Health Insurance Sharing Service database of the National Health Insurance Corporation, which stores all medical claims data, including qualification, insurance premium, long-term nursing insurance for the elderly, registration status for rare and incurable diseases, health check-ups, clinic visits, and treatment in South Korea. Survival data were collected from the Korean Statistical Information Service. BMI data were obtained from the biennial health screening programs provided by National Health Insurance Sharing Service for all Korean citizens aged > 20 years^18^. This study was approved by the Institutional Review Board of Asan Medical Center (no. S2021-1136), and informed consent was waived because of the retrospective design and use of de-identified health claims data. All methods were performed in accordance with the relevant guidelines and regulations.

### Study population

Patients with IPF were identified using the diagnostic code (J84.1) from the Korean Standard Classification of Diseases, 7th edition, and the rare intractable diseases (RID) program (Supplementary Table S1). The RID program provides medical expense support for patients with rare and incurable diseases, and registration requires strict criteria set by the National Health Insurance (NHI). Diagnosis codes in RID are highly reliable, and studies using the RID registration database have been published^19–21^. The inclusion criteria for IPF in the RID program include confirmation of the usual interstitial pneumonia pattern on a surgical lung biopsy or chest computed tomography (CT) and the exclusion of other known causes of interstitial lung disease (ILD).

**Figure 1:**
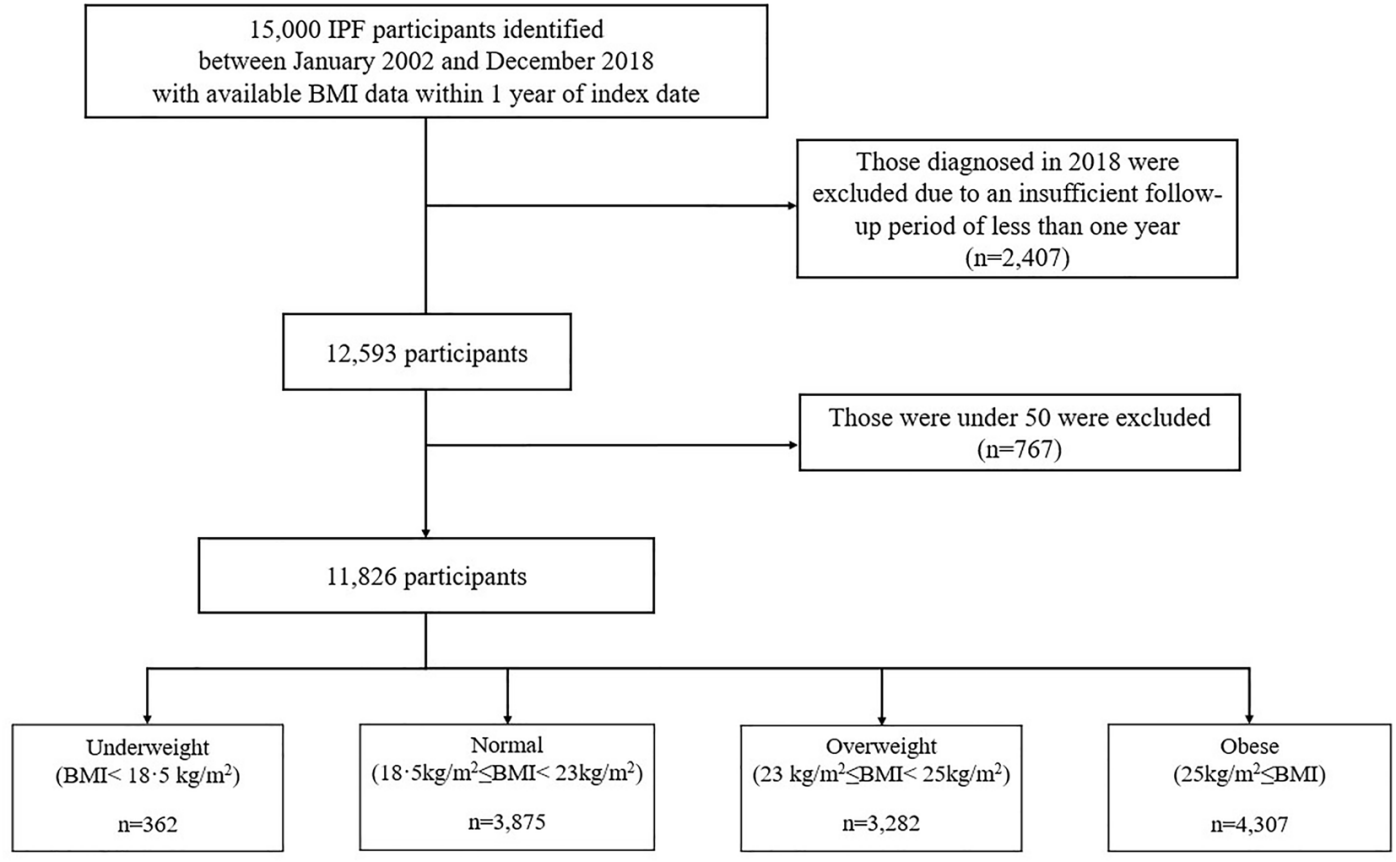
Flow diagram of the study population. *IPF* idiopathic pulmonary fibrosis, *KCD-7* Korean Standard Classification of Diseases, 7th edition, *RID* rare intractable diseases, *BMI* body mass index.

Patients who visited secondary or tertiary medical institutes between January 2002 and December 2018 with both KCD-7 (J84.1) and RID registration codes (V236) and underwent chest CT within 3 months from the index date (the first date assigned to J84.1 and V236 codes) were defined as having IPF. Among 15,000 eligible IPF patients with available BMI data within 1 year of index date, the following patients were excluded: patients who received the first IPF diagnostic code in 2018 (n = 2407; insufficient follow-up period) and those < 50 years of age (n = 767; low possibility of IPF diagnosis). Finally, 11,826 participants were included in this study (Fig. 1).

### Definition

BMI was classified according to the Asia–Pacific obesity criteria^22^: underweight (< 18.5 kg/m^2^), normal (18.5–22.9 kg/m^2^), overweight (23–24.9 kg/m^2^), and obese (≥ 25.0 kg/m^2^). The follow-up periods were calculated from the index date until the occurrence of death or censoring (December 2018). Clinical outcomes included all-cause mortality and hospitalization (all-cause and respiratory); respiratory hospitalization was defined as hospital admissions with diagnoses of respiratory system diseases classified under KCD-7 J00-J99 in addition to the IPF code (Supplementary Table S1). We identified comorbidities as repeated visits with the same disease codes within a year from the index date. Treatment data such as the at least 1-month prescription of antifibrotics (pirfenidone) and corticosteroids (oral or injectable), household income (low income was defined as the bottom 30% of the National Health Insurance premium), and type of patient’s residence (urban [Seoul and Metropolitan cities] or rural [other areas]) were collected.

### Statistical analysis

Continuous data were presented as mean ± standard deviation, whereas categorical data were presented as numbers (percentages). Differences in variables were compared using the chi-square test or analysis of variance test. Survival analysis for mortality or hospitalization was conducted using Kaplan–Meier survival curves, and between-group differences were assessed using log-rank tests. The association between BMI and clinical outcomes was assessed using the Cox proportional hazards model. BMI was analyzed as both a continuous variable and a categorized variable by using quartiles: Q1 (BMI 12.9–22 kg/m^2^), Q2 (BMI 22.1–23.9 kg/m^2^), Q3 (BMI 24.0–25.9 kg/m^2^), and Q4 (BMI 26.0–41.3 kg/m^2^). Multivariable analysis was performed after adjusting for preselected covariates, including clinical variables (age, sex, diagnosis year, Charlson comorbidity index, prescribed medication, and home oxygen use) and socioeconomic variables (type of insurance [National Health Insurance vs. medical aid], income [high vs. low], and regional type [urban vs. rural]). Moreover, after adjusting for these covariates, a spline curve HR was used to evaluate the possible non-linear relationships between BMI and clinical outcomes. On the basis of previous studies that used the National Health Insurance Sharing Service database^23,24^, the adjusted HR was calculated by using a BMI cutoff of 22.0 kg/m^2^ as reference to represent the normal value. A two-tailed *p*-value of < 0.05 was considered statistically significant. All statistical analyses were performed using SAS version 9.4 (SAS Institute, Cary, NC, USA).

## Results

### Baseline characteristics and outcomes

**Figure 2:**
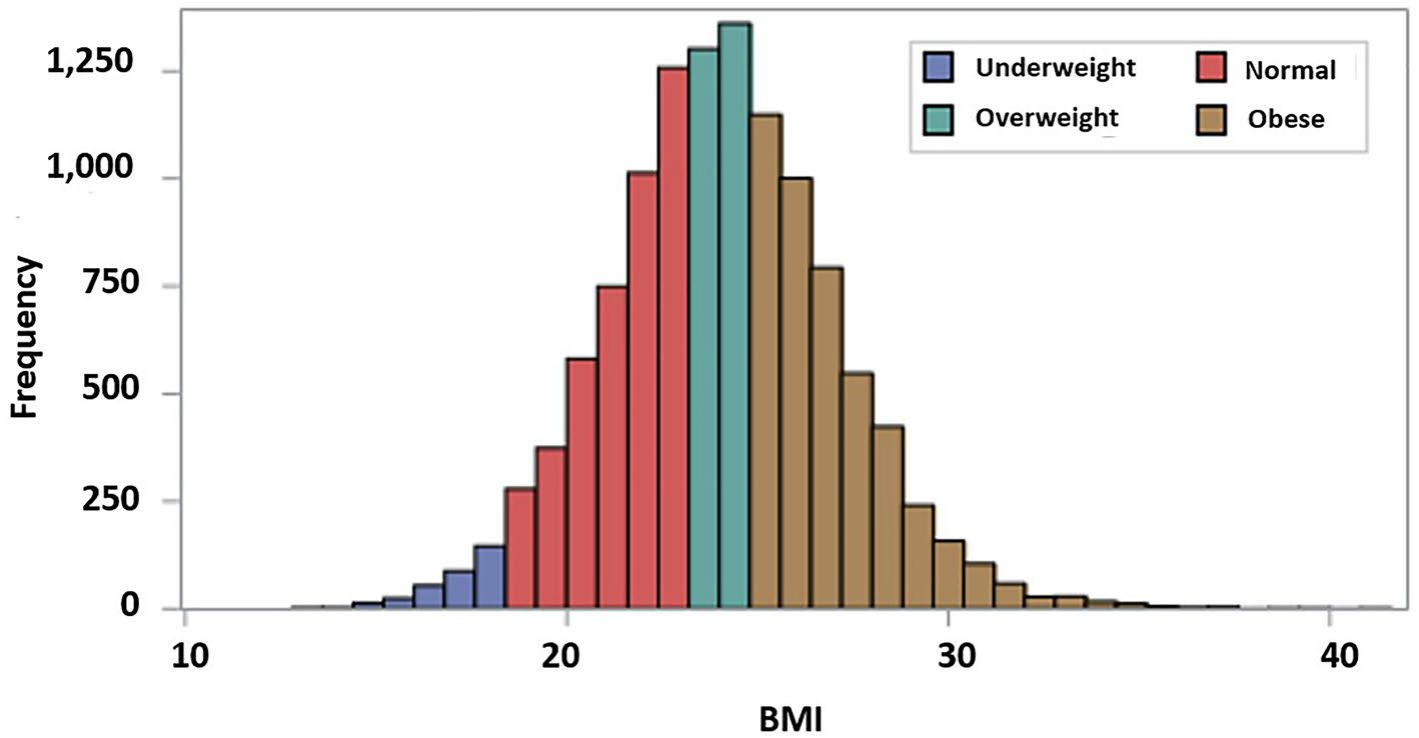
Distribution of BMI in the whole IPF cohort. IPF, idiopathic pulmonary fibrosis; BMI, body mass index.

The mean age of all patients was 68.9 years, and 73.8% of the patients were male (Table 1). Among the patients, 362 (3.1%), 3875 (32.8%), 3282 (27.8%), and 4307 (36.4%) patients were classified as underweight, normal, overweight, and obese, respectively (Fig. 2). The underweight group was older, more likely to be female, less likely to be ever-smokers, had higher Charlson comorbidity index scores, and received less treatment (pirfenidone and corticosteroid) compared with the other groups (Table 1). Furthermore, the underweight group had a shorter time to death or hospitalization (all-cause and respiratory) than other groups (Table 2).

**Table 1:** Comparison of baseline characteristics of patients with IPF according to BMI.

	Total	Underweight	Normal	Overweight	Obese	*p*-value
Number of patients	11,826	362	3875	3282	4307	
Age	68.9 ± 8.1	71.1 ± 8.5	69.7 ± 8.5	68.8 ± 8.0	68.0 ± 7.7	< 0.001
Male	8732 (73.8)	253 (69.9)	2822 (72.8)	2493 (76.0)	3164 (73.5)	0.005
Ever-smokers (n = 10,174)	4605 (45.3)	139 (42.0)	3381 (42.8)	2775 (46.9)	1718 (46.6)	< 0.001
Low household income	1757 (14.9)	61 (16.9)	568 (14.7)	488 (14.9)	640 (14.9)	0.739
Medical Aid	129 (1.1)	7 (1.9)	46 (1.2)	36 (1.1)	40 (0.9)	0.288
Comorbidity						
Lung cancer	1262 (10.7)	46 (12.7)	457 (11.8)	326 (9.9)	433 (10.1)	0.016
Diabetes mellitus	6001 (50.7)	161 (44.5)	1852 (47.8)	1648 (50.2)	2340 (54.3)	< 0.001
Dyslipidemia	7823 (66.2)	209 (57.7)	2425 (62.6)	2170 (66.1)	3019 (70.1)	< 0.001
Hypertension	6881 (58.2)	148 (40.9)	2028 (52.3)	1914 (58.3)	2791 (64.8)	< 0.001
Ischemic heart disease	3614 (30.6)	90 (24.9)	1094 (28.2)	997 (30.4)	1433 (33.3)	< 0.001
Arrhythmias	1354 (11.4)	38 (10.5)	417 (10.8)	406 (12.4)	493 (11.4)	0.189
Infection	2256 (19.1)	108 (29.8)	833 (21.5)	578 (17.6)	737 (17.1)	< 0.001
Tuberculosis	2141 (18.1)	100 (27.6)	796 (20.5)	551 (16.8)	694 (16.1)	< 0.001
NTM	97 (0.8)	10 (2.8)	47 (1.2)	25 (0.8)	15 (0.3)	< 0.001
Fungal infection	92 (0.8)	7 (1.9)	28 (0.7)	22 (0.7)	35 (0.8)	0.073
Invasive pulmonary aspergillosis	104 (0.9)	7 (1.9)	33 (0.9)	24 (0.7)	40 (0.9)	0.133
COPD	1241 (10.5)	71 (19.6)	441 (11.4)	333 (10.1)	396 (9.2)	< 0.001
Renal failure	868 (7.3)	24 (6.6)	305 (7.9)	227 (6.9)	312 (7.2)	< 0.001
CCI	1.3 ± 0.9	1.4 ± 0.9	1.3 ± 0.9	1.2 ± 0.9	1.3 ± 0.9	0.007
Treatment						
Pirfenidone	3156 (26.7)	42 (11.6)	854 (22.0)	895 (27.3)	1365 (31.7)	< 0.001
Corticosteroid	7597 (64.2)	210 (58.0)	2411 (62.2)	2148 (65.1)	2828 (65.7)	< 0.001
Home oxygen supply	137 (1.2)	1 (0.3)	39 (1.0)	37 (1.1)	60 (1.4)	0.149

Data were presented as mean ± standard deviation or number with frequency.

*IPF* idiopathic pulmonary fibrosis, *BMI* body mass index, *NTM* nontuberculous mycobacterial, *COPD* chronic obstructive lung disease, *CCI* Charlson comorbidity index.

**Table 2:** Comparison of clinical outcomes in patients with IPF according to BMI.

	Total	Underweight	Normal	Overweight	Obese	*p*-value
Number	11,826	362	3875	3282	4307	
Death						
Number of events*	5747 (48.6)	245 (67.7)	2012 (51.9)	1519 (46.3)	1971 (45.8)	< 0.001
Time to death, median years (IQR)	3.5 (0.0–17.0)	2.4 (0.0–14.1)	3.3 (0.0–16.9)	3.8 (0.0–17.0)	3.6 (0.0–17.0)	< 0.001
All-cause hospitalization						
Number of events	8.2 ± 12.1	7.8 ± 9.2	8.2 ± 11.7	8.0 ± 10.2	8.3 ± 13.8	0.689
Time to admission, median years (IQR)	0.7 (0.0–14.4)	0.4 (0.4–8.2)	0.6 (0.0–13.9)	0.8 (0.0–14.4)	0.7 (0.0–12.7)	< 0.001
Respiratory hospitalization						
Number of events	4.3 ± 11.3	4.3 ± 6.4	4.1 ± 9.0	4.1 ± 7.7	4.4 ± 14.9	0.926
Time to admission, median years (IQR)	1.6 (0.0–16.9)	1.0 (0.0–14.1)	1.5 (0.0–16.9)	1.8 (0.0–16.9)	1.6 (0.0–16.9)	< 0.001

Data were presented as mean ± standard deviation or median (interquartile range), unless otherwise indicated.

*IPF* idiopathic pulmonary fibrosis, *BMI* body mass index, *IQR* interquartile range.

*Data were presented as number (%).

### Association between BMI and mortality

**Figure 3:**
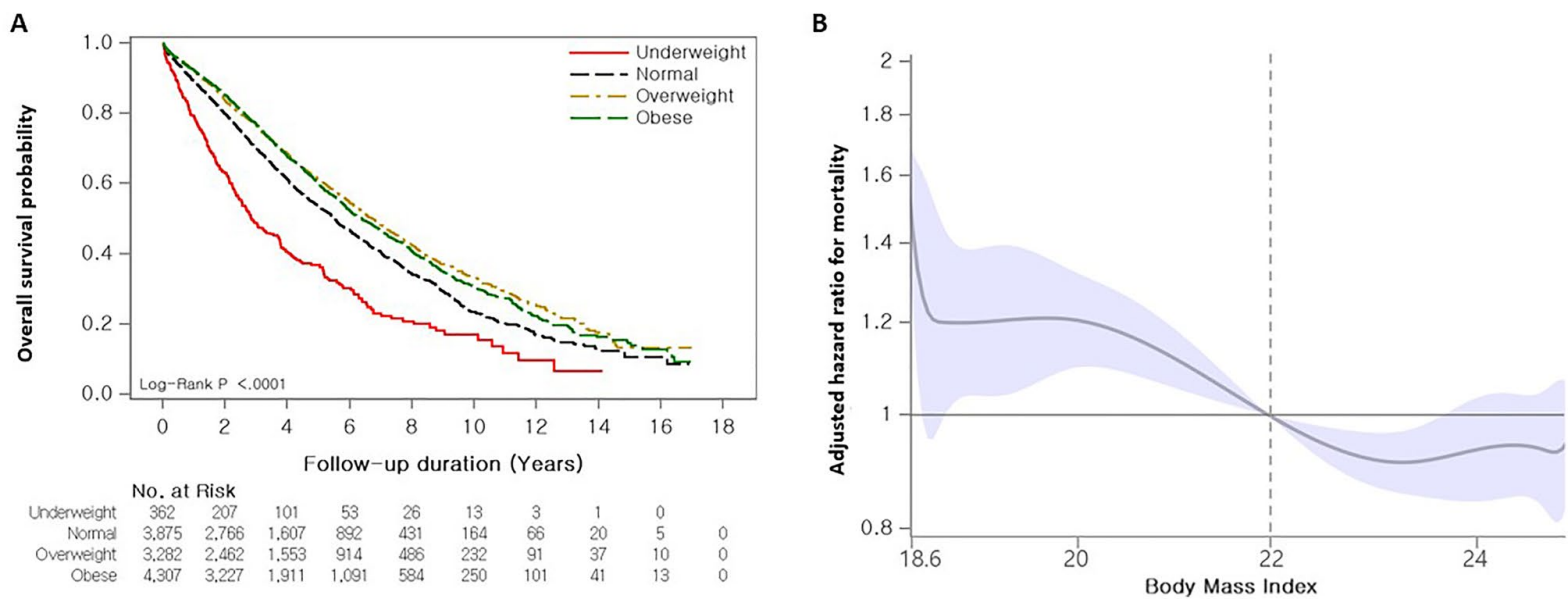
Association between BMI and mortality in patients with IPF. (**a**) Comparison of Kaplan–Meier survival curves according to BMI categories in patients with IPF. (**b**) Spline curve analysis for mortality. The spline curve hazard ratio was calculated and adjusted for covariates such as age, sex, diagnosis year, Charlson comorbidity index, medication use (steroid and pirfenidone), insurance type, residential type, and household income. A reference BMI of 22.0 kg/m^2^ was used for calculating the adjusted hazard ratio (vertical dotted line).

During the follow-up period (median: 3.5 years; interquartile range: 0.0–17.0 years), 5747 (48.6%) patients died, and the median survival period was 6.1 years (95% CI 5.9–6.2). In the Kaplan–Meier survival curve analysis, both overweight and obese groups showed better survival (median survival period: 6.7 years [overweight], 6.4 years [obese], 5.5 years [normal], 2.9 years [underweight]; *p* < 0.001]) than the other groups (Fig. 3A). There was no significant difference in survival between the overweight and obese groups (*p* > 0.999). In addition, the normal group had significantly better survival than the underweight group (*p* = 0.012).

In the unadjusted Cox analysis, being underweight was associated with increased mortality risk, whereas being overweight or obese was associated with decreased mortality risk in patients with IPF compared with those having a normal weight. In the multivariable analysis, being underweight (HR 1.538; 95% CI 1.347–1.757) and overweight (HR 0.856; 95% CI 0.801–0.916) were independently associated with mortality (Table 3). BMI levels as continuous variables were also associated with a reduced risk of mortality in patients with IPF in both unadjusted analysis (HR 0.957; 95% CI 0.948–0.966) and multivariable analysis (HR 0.978; 95% CI 0.969–0.987) (Table 3). Stratifying BMI into quartiles revealed a decreased risk of IPF mortality in all quartiles compared to Q1 (reference; BMI 12.9–22 kg/m^2^) in the multivariable analysis (Table 3).

**Table 3:** Cox proportional hazards analysis for risk factors of mortality in patients with IPF.

	Unadjusted			Multivariable		
	HR	95% CI	*p*-value	HR	95% CI	*p*-value
BMI category						
Normal	1.000			1.000		
Underweight	1.716	1.503–1.960	< 0.001	1.538	1.347–1.757	< 0.001
Overweight	0.786	0.735–0.840	< 0.001	0.856	0.801–0.916	< 0.001
Obese	0.813	0.764–0.865	< 0.001	0.943	0.886–1.004	0.065
BMI						
Continuous	0.957	0.948–0.966	< 0.001	0.978	0.969–0.987	< 0.001
Q1	1.000			1.000		
Q2	0.700	0.652–0.752	< 0.001	0.766	0.713–0.823	< 0.001
Q3	0.685	0.638–0.737	< 0.001	0.802	0.746–0.862	< 0.001
Q4	0.719	0.669–0.773	< 0.001	0.857	0.796–0.922	< 0.001

BMI was categorized into four groups on the basis of quartiles: Q1 (BMI 12.9–22 kg/m^2^), Q2 (BMI 22.1–23.9 kg/m^2^), Q3 (BMI 24.0–25.9 kg/m^2^), and Q4 (BMI 26.0–41.3 kg/m^2^). A multivariable model was used that was adjusted for age, sex, diagnosis year, Charlson comorbidity index, medication use (steroid, pirfenidone), insurance type, residential type, and household income.

*IPF* idiopathic pulmonary fibrosis, *HR* hazard ratio, *CI* confidence interval, *BMI* body mass index.

In the spline HR curve adjusted by clinical and socioeconomic covariates, a non-linear relationship between BMI and mortality was observed in patients with IPF (Fig. 3B) (Supplementary Table S2). Those with BMI < 22.0 kg/m^2^ had a significantly increased mortality risk, whereas those with a 22.0 < BMI ≤ 23.6 kg/m^2^ had a decreased risk of mortality. For patients with a BMI > 23.6 kg/m^2^, the mortality risk did not differ from the reference (BMI 22.0 kg/m^2^).

### Association between BMI and hospitalization

**Figure 4:**
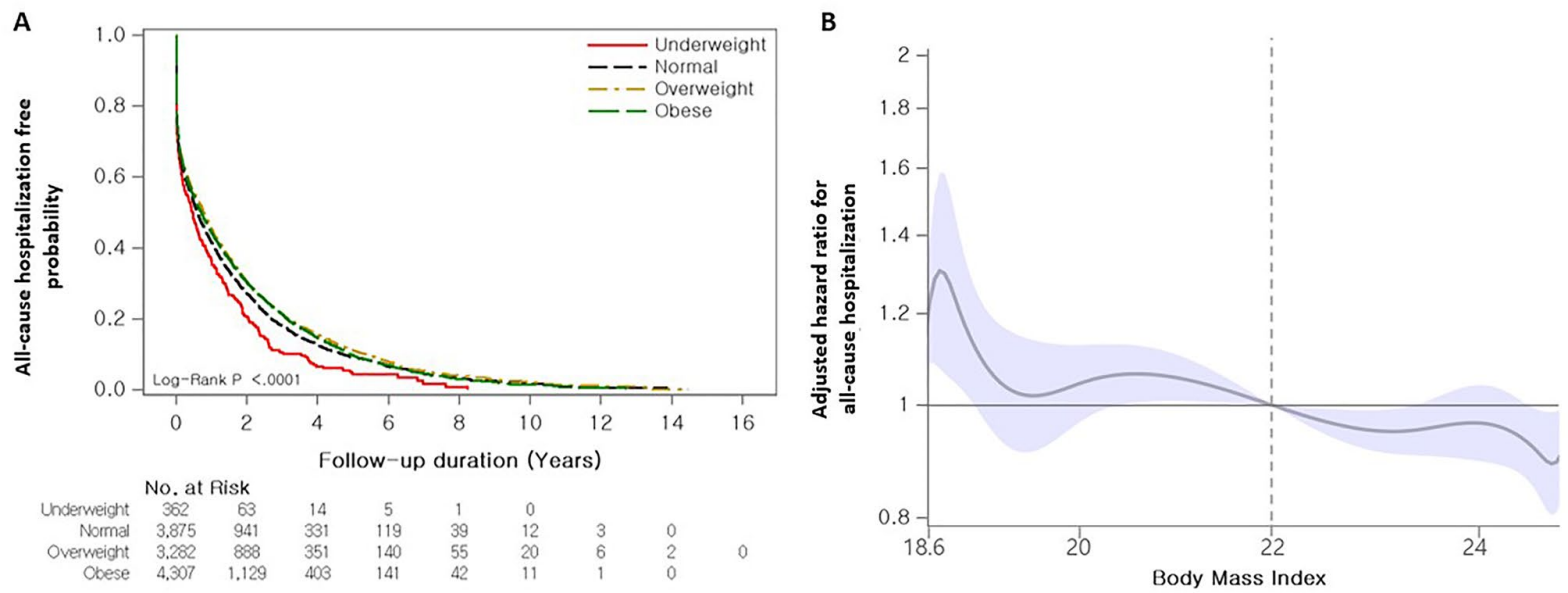
Association between BMI and all-cause hospitalization in patients with IPF. (**a**) Comparison of all-cause hospitalization-free survival curves according to BMI categories in patients with IPF. (**b**) Spline curve analysis for all-cause hospitalization. The spline curve hazard ratio was calculated and adjusted for covariates such as age, sex, diagnosis year, Charlson comorbidity index, medication use (steroid and pirfenidone), insurance type, residential type, and household income. A reference BMI of 22.0 kg/m^2^ was used for calculating the adjusted HR (vertical dotted line).

**Figure 5:**
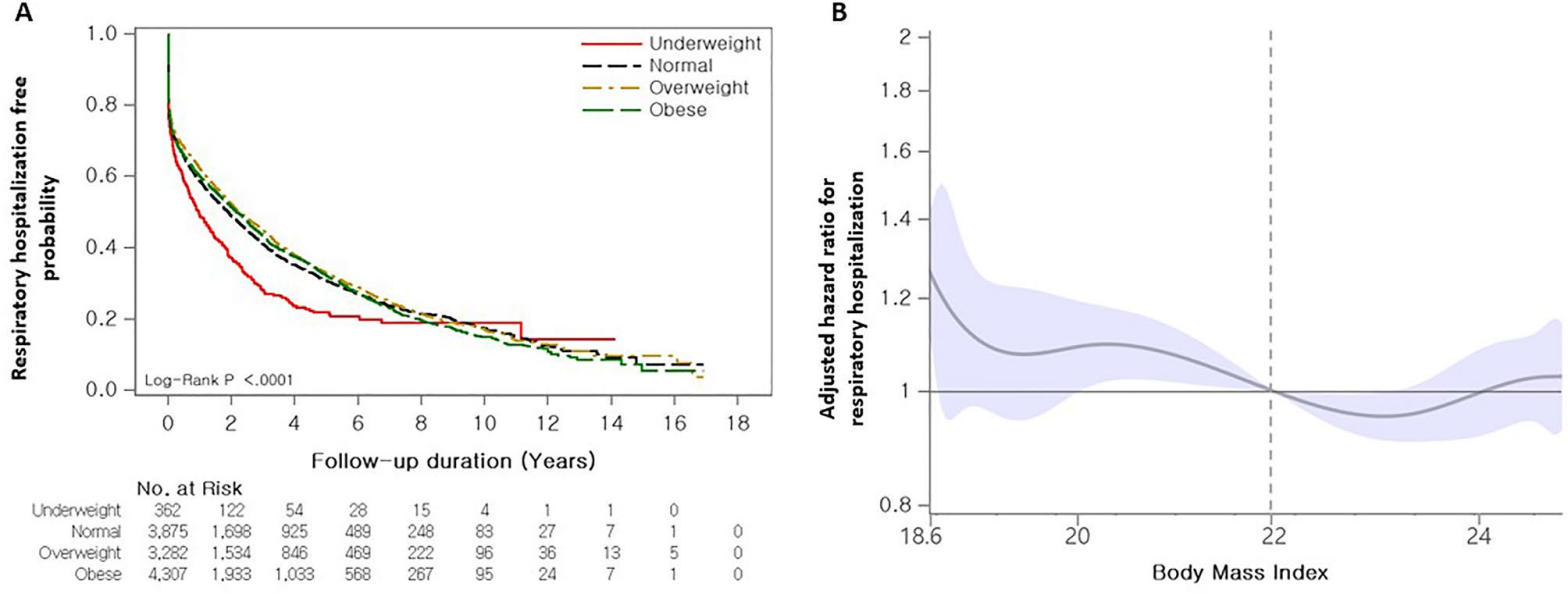
Association between BMI and respiratory hospitalization in patients with IPF. (**a**) Comparison of respiratory hospitalization-free survival curves according to BMI categories in patients with IPF. (**b**) Spline curve analysis for respiratory hospitalization. The spline curve hazard ratio was calculated and adjusted for covariates such as age, sex, diagnosis year, Charlson comorbidity index, medication use (steroid and pirfenidone), insurance type, residential type, and household income. A reference BMI of 22.0 kg/m^2^ was used for calculating the adjusted HR (vertical dotted line).

The median hospitalization-free survival time for all-cause and respiratory hospitalization was 0.7 years (95% CI 0.6–0.7 years) and 2.1 years (95% CI 1.9–2.2 years), respectively. The overweight and obese groups exhibited better all-cause hospitalization-free survival (median survival period: 0.7 years [overweight], 0.8 years [obese], 0.6 years [normal], 0.5 years [underweight], *p* < 0.001) than the other groups, but no differences were observed between the overweight and obese groups (*p* = 0.960) or between the normal and underweight groups (*p* = 0.534) (Fig. 4A). In terms of respiratory hospitalization, the obese group showed better hospitalization-free survival (median survival period: 2.2 years [obese] vs. 1.0 years [underweight], *p* < 0.001) than the underweight group; however, no differences were observed between the other groups (2.2 years [overweight], 1.9 years [normal]) (Fig. 5A).

The unadjusted Cox analysis revealed that being underweight was associated with an increased risk of all-cause hospitalization, whereas being overweight and obese were associated with a decreased risk of hospitalization compared with having a normal weight (as reference) (Table 4). In the multivariable analysis, being underweight (HR 1.265; 95% CI 1.104–1.449), overweight (HR 0.887; 95% CI 0.834–0.943), and obese (HR 0.925; 95% CI 0.873–0.980) were independently associated with a risk of all-cause hospitalization compared with having a normal weight (Table 4). Similar results were also observed for respiratory hospitalization (Table 4). BMI as a continuous variable was associated with a reduced risk of all-cause hospitalization (HR 0.985; 95% CI 0.977–0.993), but it was not associated with respiratory hospitalization in the multivariable analysis adjusted for clinical and socioeconomic variables (Table 4). When BMI was classified into quartiles, all quartiles showed a decreased risk of all-cause hospitalization compared with Q1 (HR for Q2 [95% CI] 0.851 [0.796–0.910]; Q3: 0.829 [0.775–0.887]; Q4: 0.873 [0.815–0.934]) in the multivariable analysis (Table 4). Similar results were observed for respiratory hospitalizations (HR for Q2 [95% CI] 0.871 [0.819–0.927]; Q3: 0.907 [0.852–0.964]), except for Q4 (HR: 0.962; 95% CI: 0.904–1.023).

**Table 4:** Cox proportional hazards analysis for risk factors of hospitalization in patients with IPF.

	All-cause						Respiratory					
	Unadjusted			Multivariable			Unadjusted			Multivariable		
	HR	95% CI	*p*-value	HR	95% CI	*p*-value	HR	95% CI	*p*-value	HR	95% CI	*p*-value
BMI category												
Normal	1.000			1.000			1.000			1.000		
Underweight	1.375	1.201–1.575	< 0.001	1.265	1.104–1.449	< 0.001	1.266	1.118–1.434	< 0.001	1.232	1.088–1.395	0.001
Overweight	0.843	0.793–0.896	< 0.001	0.887	0.834–0.943	< 0.001	0.941	0.890–0.995	0.0323	0.953	0.901–1.008	0.092
Obese	0.857	0.809–0.908	< 0.001	0.925	0.873–0.980	0.008	0.982	0.932–1.035	0.498	0.998	0.947–1.051	0.933
BMI												
Continuous	0.973	0.965–0.981	< 0.001	0.985	0.977–0.993	< 0.001	0.995	0.987–1.002	0.170	0.990	0.974–1.006	0.223
Q1	1.000			1.000			1.000			1.000		
Q2	0.805	0.753–0.860	< 0.001	0.851	0.796–0.910	< 0.001	0.857	0.806–0.911	< 0.001	0.871	0.819–0.927	< 0.001
Q3	0.763	0.714–0.816	< 0.001	0.829	0.775–0.887	< 0.001	0.885	0.832–0.941	< 0.001	0.907	0.852–0.964	0.002
Q4	0.796	0.744–0.852	< 0.001	0.873	0.815–0.934	< 0.001	0.943	0.887–1.003	0.064	0.962	0.904–1.023	0.216

BMI was categorized into four groups on the basis of quartiles: Q1 (BMI 12.9–22 kg/m^2^), Q2 (BMI 22.1–23.9 kg/m^2^), Q3 (BMI 24.0–25.9 kg/m^2^), and Q4 (BMI 26.0–41.3 kg/m^2^). A multivariable model was used that was adjusted for age, sex, diagnosis year, Charlson comorbidity index, medication use (steroid, pirfenidone), insurance type, residential type, and household income.

*IPF* idiopathic pulmonary fibrosis, *HR* hazard ratio, *CI* confidence interval, *BMI* body mass index.

In the spline HR curve analysis using a BMI of 22.0 kg/m^2^ as a reference, the risk of all-cause hospitalization increased at a BMI < 22.0 kg/m^2^ but decreased at a BMI > 22.0 kg/m^2^ (Fig. 4b) (Supplementary Table S3). The risk for respiratory hospitalization also tended to decrease at a BMI > 22.0 kg/m^2^, although this was not statistically significant. However, for a BMI < 22 kg/m^2^, the risk of respiratory hospitalization significantly increased at a BMI of 18.4 kg/m^2^ (HR 1.312; 95% CI 1.150–1.497), but it showed an insignificant association with other BMI values (Fig. 5B) (Supplementary Table S4).

## Discussion

This large-scale, nationwide, population-based study investigated the association between BMI and the clinical outcomes of IPF by using the claims database. We found that being underweight was associated with increased risk of mortality and hospitalization, whereas obesity and overweight were inversely associated with increased risk of mortality and hospitalization. Spline curve analysis revealed the non-linear association between BMI and prognosis. Specifically, a BMI below 22.0 kg/m^2^ was associated with an increased risk of death or hospitalization, whereas a BMI above 22.0 kg/m^2^ was associated with a decreased risk of these outcomes in patients with IPF.

Our study showed that a lower BMI was associated with worse prognosis, whereas a higher BMI was associated with better prognosis in patients with IPF. This is in line with previous studies that have shown increased mortality risk in patients with IPF who have lower BMI^6,7,14–16^. In a retrospective IPF cohort (n = 197), Alakhras et al. showed that a lower BMI was associated with increased mortality (HR 0.86; 95% CI 0.79–0.94) in a multivariable Cox analysis adjusted for gender, biopsy-confirmed diagnosis, forced vital capacity, diffusing capacity for carbon monoxide, and minimum oxygen saturation during exercise test^6^. Wright et al. studied 104 patients with IPF and found that a BMI ≤ 25.0 kg/m^2^ was independently associated with increased risk of 12-month mortality (odds ratio [OR] 3.3; 95% CI 1.44–7.59) in the multivariable logistic analysis adjusted for carbon monoxide and home oxygen use^15^. Awano et al. used the Japanese Diagnosis Procedure Combination database to analyze patients with IPF who experienced acute exacerbation (AE) (n = 14,783) and found that patients who were underweight (< 18.5 kg/m^2^) or obese (30.0 kg/m^2^) had increased (OR 1.25; 95% CI 1.10–1.42) or decreased (OR 0.71; 95% CI 0.54–0.84) risk of in-hospital mortality, respectively, in a multivariable logistic analysis^8^. Similar results were found in patients with ILD^14^. In a multicenter study of patients with ILD (n = 1786; IPF = 34%), Comes et al. reported that a higher BMI was associated with decreased risk of 1-year mortality (HR 0.93; 95% CI 0.90–0.97) in a multivariable Cox analysis adjusted for the ILD–gender, age, and physiology index^14^. These collective findings further support the importance of BMI as a prognostic factor in IPF.

In this study, being underweight was an independent risk factor for poor clinical outcomes. Although the underlying mechanisms of this effect are not fully understood, patients with advanced chronic diseases often experience unintended weight loss because of loss of appetite, mental illness, or hormonal changes. Previous studies have also suggested that hormones involved in body weight regulation, such as leptin and adiponectin, may be association with the progression of fibrosis^25–27^. Adiponectin, one of the adipokines derived from adipocytes, is negatively correlated with BMI in patients with IPF^25^ and has been shown to stimulate collagen production^26^ and the differentiation of monocytes into fibroblasts^27^. These findings suggest that increased adiponectin in underweight patients with IPF may contribute to the progression of IPF. In the general population, obesity and being overweight are associated with increased mortality risk, possibly, because of immune system dysfunction^28^, disruption of lung physiology^29^, and predisposed comorbidities^30^. However, in many chronic lung or cardiovascular diseases, a protective effect of adipose tissue, known as the “obesity paradox,” has been reported^31–35^. Moreover, obesity may offer a metabolic buffer that can be utilized in times of stress or illness, thus potentially leading to better outcomes^36^. In fact, the “obesity paradox” is particularly evident in older individuals and those with advanced stages of the disease^37,38^. In addition, obese individuals are more likely to have comorbidities^30^, which may lead to an earlier diagnosis of IPF because of increased medical visits.

Our study added to the existing literature by revealing a non-linear relationship between BMI and mortality in patients with IPF. Previous studies suggested an association between BMI and IPF mortality without investigating non-linear associations^6,13,14^. Mazen et al. studied 197 patients with IPF and found a significant association between BMI and mortality (HR 0.86; 95% CI 0.79–0.94) in a multivariable Cox analysis adjusted for clinical variables^6^. Comes et al. also reported a significant association between BMI and 1-year mortality (HR 0.93; 95% CI 0.90–0.97) in patients with ILD (n = 1786; IPF = 34%) in a multivariable Cox analysis adjusting for the ILD–gender, age, and physiology index^14^. By contrast, in this study, Cox proportional analysis for mortality showed that the HR of mortality was the lowest in Q2. Additionally, the spline HR curve analysis showed that the inverse relationship between BMI and mortality was insignificant above a certain point in BMI (23.6 kg/m^2^). These results suggest that maintaining BMI in the optimal range may be beneficial for improving prognosis in patients with IPF.

Our IPF cohort had a lower proportion of underweight individuals (3.1%) and a relatively higher proportion of overweight (27.8%) and obese individuals (36.4%) compared with the general population or patients with chronic obstructive pulmonary disease in South Korea. According to the 2022 Korea National Health and Nutrition Examination Survey for the general population (n = 6265), the percentages of underweight, normal, overweight, and obese individuals were 11.6%, 35.8%, 20.0% and 32.6%, respectively (https://knhanes.kdca.go.kr/). In addition, an analysis of the Korean COPD Subtype Study cohort (n = 1462 aged 40 years or older) showed that 10.7% were underweight, 42.4% were normal weight, 21.4% were overweight and 25.5% were obese^39^. The lower prevalence of underweight and higher rates of overweight and obesity in IPF patients may be due to treatment effects such as steroids, reduced physical activity due to respiratory symptoms, or older age compared to the other population.

Our study has some limitations. First, given that this study was based on data from the claims database, we were unable to obtain sufficient information, such as lung function. Instead, we adjusted for other clinical variables that are known to affect the prognosis of IPF, including age, sex, Charlson comorbidity index, medication, and home oxygen use, in the multivariable analysis. Second, considering that we included participants who underwent general health check-ups to collect BMI data, individuals with more health concerns or specific socioeconomic status could be included^40^. However, it is worth noting that national health screening programs supported by the government typically attract a diverse range of participants, including those from low-income backgrounds. Third, we defined IPF cases by using IPF diagnostic codes; therefore, the accuracy of diagnosis in our study may be lower than that in other studies that used medical records. To reduce this limitation, both IPF and RID registration codes were used to define IPF cases in this study. Prospective studies are needed to confirm our results. Despite these limitations, our study demonstrated an association between BMI and prognosis in a large population, as well as a non-linear relationship in IPF.

Our results suggest that BMI is associated with clinical outcomes in patients with IPF. Further studies are needed to determine whether BMI derangement drives prognosis or simply reflects disease severity.

### Supplementary Information

Supplementary Tables.

## Data Availability

All the supporting data is provided as supplementary files.
