# Supplementary material for: Body mass index is associated with clinical outcomes in idiopathic pulmonary fibrosis
Source: Sci Rep. 2024 May 24;14:11921. doi: 10.1038/s41598-024-62572-4 (PMC11126411; doi:10.1038/s41598-024-62572-4)
Supplement: Supplementary file 1 — Supplementary Tables. [file 41598_2024_62572_MOESM1_ESM.docx]

**Body mass index is associated with clinical outcomes in idiopathic pulmonary fibrosis**

Hee-Young Yoon^1^, Hoseob Kim^2^, Yoonjong Bae^2^, Jin Woo Song^3^

*^1^Division of Allergy and Respiratory Diseases, Soonchunhyang University Seoul Hospital, Seoul, Republic of Korea*

*^2^Department of Data Science, Hanmi Pharm. Co., Ltd, Seoul, Republic of Korea*

*^3^Department of Pulmonary and Critical Care Medicine, Asan Medical Center, University of Ulsan College of Medicine, Seoul, Republic of Korea*

**Table S1**. Diagnostic code for respiratory system disease

| KCD code | description |
| --- | --- |
| J00-J06 | Acute upper respiratory infections |
| J10-J18 | Influenza and pneumonia |
| J20-J22 | Other acute lower respiratory infections |
| J30-J39 | Other diseases of upper respiratory tract |
| J40-J47 | Chronic lower respiratory diseases |
| J60-J70 | Lung diseases due to external agents |
| J80-J84 | Other respiratory diseases principally affecting the interstitium |
| J84.1 | Other interstitial pulmonary diseases with fibrosis |
| J85-J86 | Suppurative and necrotic conditions of lower respiratory tract |
| J90-J94 | Other pleural conditions |
| J95-J99 | Other disorders of the respiratory system |

KCD = Korean Standard Classification of Diseases

**Table S2**. Adjusted hazard ratio calculated by spline curve analysis for all-cause mortality in patients with IPF

| BMI | Adjusted HR | 95% CI | | p-value |
| --- | --- | --- | --- | --- |
|  |  | Lower | Upper |  |
| 18.4 | 1.617 | 1.413 | 1.852 | 0.000 |
| 18.6 | 1.328 | 1.077 | 1.639 | 0.008 |
| 18.8 | 1.200 | 0.972 | 1.482 | 0.090 |
| 19 | 1.198 | 1.032 | 1.391 | 0.018 |
| 19.2 | 1.200 | 1.038 | 1.386 | 0.013 |
| 19.4 | 1.203 | 1.038 | 1.395 | 0.014 |
| 19.6 | 1.207 | 1.049 | 1.388 | 0.008 |
| 19.8 | 1.209 | 1.070 | 1.367 | 0.002 |
| 20 | 1.208 | 1.089 | 1.339 | 0.000 |
| 20.2 | 1.202 | 1.099 | 1.314 | 0.000 |
| 20.4 | 1.190 | 1.097 | 1.290 | 0.000 |
| 20.6 | 1.173 | 1.087 | 1.265 | 0.000 |
| 20.8 | 1.152 | 1.075 | 1.235 | 0.000 |
| 21 | 1.129 | 1.061 | 1.201 | 0.000 |
| 21.2 | 1.103 | 1.047 | 1.163 | 0.000 |
| 21.4 | 1.077 | 1.034 | 1.122 | 0.000 |
| 21.6 | 1.050 | 1.022 | 1.080 | 0.001 |
| 21.8 | 1.025 | 1.010 | 1.039 | 0.001 |
| 22 | 1.000 | 1.000 | 1.000 | reference |
| 22.2 | 0.977 | 0.964 | 0.991 | 0.001 |
| 22.4 | 0.957 | 0.933 | 0.982 | 0.001 |
| 22.6 | 0.940 | 0.906 | 0.975 | 0.001 |
| 22.8 | 0.926 | 0.885 | 0.969 | 0.001 |
| 23 | 0.917 | 0.870 | 0.966 | 0.001 |
| 23.2 | 0.912 | 0.860 | 0.966 | 0.002 |
| 23.4 | 0.912 | 0.854 | 0.973 | 0.005 |
| 23.6 | 0.917 | 0.849 | 0.990 | 0.026 |
| 23.8 | 0.925 | 0.845 | 1.012 | 0.088 |
| 24 | 0.933 | 0.844 | 1.033 | 0.180 |
| 24.2 | 0.940 | 0.847 | 1.043 | 0.245 |
| 24.4 | 0.942 | 0.852 | 1.042 | 0.246 |
| 24.6 | 0.938 | 0.845 | 1.041 | 0.229 |
| 24.8 | 0.930 | 0.809 | 1.069 | 0.306 |
| 25 | 1.007 | 0.938 | 1.082 | 0.8417 |

IPF = idiopathic pulmonary fibrosis; BMI = body mass index; HR = hazard ratio; CI = confidence interval

The spline curve hazard ratio was calculated and adjusted for covariates such as age, sex, diagnosis year, Charlson comorbidity index, medication use (steroid and pirfenidone), insurance type, residential type, and household income. A reference BMI of 22.0 kg/m² was used for calculating the adjusted HR

**Table S3.** Adjusted hazard ratio calculated by spline curve analysis for all-cause hospitalization in patients with IPF

| BMI | Adjusted HR | 95% CI | | p-value |
| --- | --- | --- | --- | --- |
|  |  | Lower | Upper |  |
| 18.4 | 1.134 | 1.011 | 1.271 | 0.032 |
| 18.6 | 1.284 | 1.086 | 1.518 | 0.003 |
| 18.8 | 1.239 | 1.047 | 1.466 | 0.013 |
| 19 | 1.124 | 0.999 | 1.265 | 0.052 |
| 19.2 | 1.059 | 0.945 | 1.186 | 0.324 |
| 19.4 | 1.027 | 0.915 | 1.153 | 0.648 |
| 19.6 | 1.019 | 0.913 | 1.138 | 0.732 |
| 19.8 | 1.026 | 0.932 | 1.130 | 0.597 |
| 20 | 1.040 | 0.960 | 1.127 | 0.336 |
| 20.2 | 1.053 | 0.984 | 1.128 | 0.137 |
| 20.4 | 1.062 | 0.998 | 1.129 | 0.058 |
| 20.6 | 1.064 | 1.005 | 1.127 | 0.032 |
| 20.8 | 1.063 | 1.009 | 1.120 | 0.023 |
| 21 | 1.058 | 1.009 | 1.108 | 0.019 |
| 21.2 | 1.049 | 1.009 | 1.092 | 0.017 |
| 21.4 | 1.039 | 1.007 | 1.071 | 0.017 |
| 21.6 | 1.026 | 1.005 | 1.048 | 0.016 |
| 21.8 | 1.013 | 1.003 | 1.024 | 0.015 |
| 22 | 1.000 | 1.000 | 1.000 | reference |
| 22.2 | 0.987 | 0.977 | 0.997 | 0.014 |
| 22.4 | 0.975 | 0.956 | 0.995 | 0.014 |
| 22.6 | 0.965 | 0.938 | 0.993 | 0.013 |
| 22.8 | 0.957 | 0.924 | 0.991 | 0.014 |
| 23 | 0.952 | 0.914 | 0.991 | 0.015 |
| 23.2 | 0.950 | 0.909 | 0.993 | 0.022 |
| 23.4 | 0.952 | 0.906 | 1.000 | 0.048 |
| 23.6 | 0.957 | 0.904 | 1.013 | 0.129 |
| 23.8 | 0.963 | 0.902 | 1.029 | 0.264 |
| 24 | 0.966 | 0.897 | 1.040 | 0.359 |
| 24.2 | 0.963 | 0.892 | 1.039 | 0.327 |
| 24.4 | 0.949 | 0.882 | 1.022 | 0.165 |
| 24.6 | 0.923 | 0.855 | 0.997 | 0.042 |
| 24.8 | 0.891 | 0.804 | 0.987 | 0.027 |
| 25 | 0.986 | 0.936 | 1.039 | 0.606 |

IPF = idiopathic pulmonary fibrosis; BMI = body mass index; HR = hazard ratio; CI = confidence interval

The spline curve hazard ratio was calculated and adjusted for covariates such as age, sex, diagnosis year, Charlson comorbidity index, medication use (steroid and pirfenidone), insurance type, residential type, and household income. A reference BMI of 22.0 kg/m² was used for calculating the adjusted HR

**Table S4.** Adjusted hazard ration calculated by spline curve analysis for respiratory hospitalization in patients with IPF

| BMI | Adjusted HR | 95% CI | | p-value |
| --- | --- | --- | --- | --- |
|  |  | Lower | Upper |  |
| 18.4 | 1.336 | 1.165 | 1.531 | 0.000 |
| 18.6 | 1.257 | 1.029 | 1.536 | 0.025 |
| 18.8 | 1.210 | 0.989 | 1.481 | 0.064 |
| 19 | 1.198 | 1.038 | 1.383 | 0.014 |
| 19.2 | 1.186 | 1.032 | 1.362 | 0.016 |
| 19.4 | 1.174 | 1.019 | 1.352 | 0.026 |
| 19.6 | 1.161 | 1.016 | 1.327 | 0.028 |
| 19.8 | 1.149 | 1.022 | 1.291 | 0.020 |
| 20 | 1.135 | 1.030 | 1.252 | 0.011 |
| 20.2 | 1.122 | 1.032 | 1.220 | 0.007 |
| 20.4 | 1.108 | 1.027 | 1.195 | 0.008 |
| 20.6 | 1.093 | 1.019 | 1.172 | 0.013 |
| 20.8 | 1.078 | 1.011 | 1.150 | 0.022 |
| 21 | 1.064 | 1.004 | 1.127 | 0.035 |
| 21.2 | 1.050 | 1.000 | 1.102 | 0.051 |
| 21.4 | 1.036 | 0.997 | 1.076 | 0.068 |
| 21.6 | 1.023 | 0.997 | 1.050 | 0.085 |
| 21.8 | 1.011 | 0.998 | 1.024 | 0.101 |
| 22 | 1.000 | 1.000 | 1.000 | reference |
| 22.2 | 0.990 | 0.978 | 1.003 | 0.127 |
| 22.4 | 0.982 | 0.958 | 1.006 | 0.137 |
| 22.6 | 0.975 | 0.942 | 1.009 | 0.145 |
| 22.8 | 0.969 | 0.929 | 1.012 | 0.153 |
| 23 | 0.966 | 0.920 | 1.014 | 0.166 |
| 23.2 | 0.965 | 0.914 | 1.018 | 0.193 |
| 23.4 | 0.965 | 0.909 | 1.026 | 0.253 |
| 23.6 | 0.968 | 0.902 | 1.038 | 0.358 |
| 23.8 | 0.969 | 0.892 | 1.052 | 0.448 |
| 24 | 0.965 | 0.880 | 1.058 | 0.453 |
| 24.2 | 0.955 | 0.868 | 1.050 | 0.339 |
| 24.4 | 0.934 | 0.852 | 1.023 | 0.142 |
| 24.6 | 0.900 | 0.818 | 0.990 | 0.031 |
| 24.8 | 0.863 | 0.760 | 0.979 | 0.023 |
| 25 | 0.971 | 0.910 | 1.037 | 0.379 |

IPF = idiopathic pulmonary fibrosis; BMI = body mass index; HR = hazard ratio; CI = confidence interval

The spline curve hazard ratio was calculated and adjusted for covariates such as age, sex, diagnosis year, Charlson comorbidity index, medication use (steroid and pirfenidone), insurance type, residential type, and household income. A reference BMI of 22.0 kg/m² was used for calculating the adjusted HR
